# A Comprehensive Review of 3D Imaging and Printing in Proximal Humerus Fractures and Sequelae

**DOI:** 10.3390/jcm14217711

**Published:** 2025-10-30

**Authors:** Roberto de Giovanni, Martina Coppola, Valentina Rossi, Massimo Mariconda, Andrea Cozzolino

**Affiliations:** Department of Public Health, Naples University Hospital “Federico II”, Via Pansini 5, 80131 Naples, Italyandrea.cozzolino2@unina.it (A.C.)

**Keywords:** fracture sequelae, 3D printing, proximal humerus fracture

## Abstract

Proximal humerus fractures are common and complex; despite advances, malunion, nonunion, and osteonecrosis remain concerns. Three-dimensional (3D) imaging/printing has emerged to improve classification, planning, and execution, especially in displaced patterns. Methods: Multiple databases have been searched using predefined terms (“proximal humerus fractures/sequelae”, “three-dimensional”, and “3D printing”). Inclusion criteria targeted human longitudinal studies (retrospective/prospective) on 3D-assisted fracture or sequela management; expert opinion, prior reviews, and letters to editors were excluded. Data extracted included the design, the level of evidence (LoE), the sample size, 3D application (diagnostic, planning, intraoperative, and combined), outcomes, follow-up, and complications. Results: Nineteen studies were included (fourteen fractures and five sequelae; 636 and 28 patients, respectively). In fractures, 3D imaging was used chiefly for preoperative planning (57.1%) and diagnostic support (35.7%); no intraoperative PSI was reported. In sequelae, intraoperative/PSI dominated (100%), with planning in 80% and combined uses in 80%. Fracture studies were mostly retrospective (50.0%; LoE III 78.6%), while all sequelae were LoE IV–V (60% of case reports). Standardized outcomes were reported in 42.1% of studies; follow-up was available in 42.1% (means ≈ 18 months). Complications occurred in 14.3% of fracture studies and in none of the sequelae. Conclusions: Three-dimensional printing is primarily applied for planning in fractures and intraoperative guidance in sequelae. While feasibility and potential perioperative benefits are evident, small heterogeneous cohorts and limited outcome reporting warrant larger prospective studies with standardized endpoints.

## 1. Introduction

Proximal humerus fractures (PHFs) represent a significant orthopedic challenge, frequently resulting from falls in older adults or high-energy trauma in younger individuals [1]. These fractures are the fourth most common osteoporotic fracture, with a higher incidence in older women [2]. Given their prevalence, especially among the elderly population, these injuries often necessitate complex surgical interventions that often include the need for joint replacement [3], with estimates suggesting that 10–15% require operative management [4]. The intricate anatomy of the proximal humerus and the wide range of fracture patterns make accurate classification and preoperative planning crucial for successful outcomes [5]. Jabran et al. showed through a systematic biomechanical analysis that factors such as implant configuration, screw placement, and overall construct stiffness have a major impact on the stability of proximal humerus fixation, illustrating how mechanically demanding these fractures can be, thus helping to explain why virtual planning and modeling are increasingly seen as valuable tools to support more precise and reliable surgical strategies [1]. Indeed, despite advancements in surgical techniques, complications such as malunion, nonunion, and osteonecrosis remain significant concerns, underscoring the need for improved methods in surgical planning and execution in order to avoid the need to manage significant bone loss in a revision setting, which could be difficult to deal with even with advanced techniques [6]. Consequently, three-dimensional (3D) imaging and printing technologies have emerged as promising tools to enhance the precision of fracture classification and facilitate more effective preoperative planning, particularly for displaced fractures [2]. A similar, 3D-oriented approach for the treatment of femoral head fractures has been successfully applied [7], and indications for implementing this technology are growing daily [8]. Despite these advancements, it is important to acknowledge that the widespread adoption of 3D printing in proximal humerus fracture management is still in its early stages: challenges such as the cost of technology [8], the need for specialized training, and integration into existing workflows need to be addressed for broader clinical implementation regarding proximal humerus fractures. Such workflows have been implemented in the treatment of different anatomical districts and can be seen as part of the diagnostic and treatment algorithm [9]. 

In addition to acute fracture management, 3D technology has gained increasing attention in the treatment of post-traumatic deformities and malunions of the proximal humerus. The sequelae of proximal humerus fractures (PHFSs) tend to manifest with a combination of distorted bony anatomy, compromised bone quality, and altered soft-tissue mechanics [5]. Patients often present with persistent pain, reduced shoulder range of motion, tuberosity malposition or resorption, metaphyseal bone loss, varus or valgus malalignment, and long-standing rotator cuff dysfunction. Anatomical distortion may involve the humeral head, tuberosities, or diaphysis and is frequently compounded by chronic changes including muscle retraction, joint stiffness, and secondary osteoarthritis [10]. Because each case often reflects a unique pattern of healing failure, soft-tissue contracture, and altered load transfer, surgical reconstruction becomes considerably more complex than treatment of an acute fracture [5]. In this context, patient-specific planning and instrumentation enable surgeons to simulate corrective osteotomies preoperatively, to optimize implant selection and positioning, and to achieve more accurate realignment. This approach has been shown to enhance the precision of both osteotomy execution and prosthesis implantation, ultimately supporting better anatomical restoration and functional recovery in revision settings [10]. Nonetheless, the recent literature highlights the growing role of three-dimensional printing in complex periarticular fracture management, with a particular emphasis on its ability to enhance preoperative planning and surgical precision through patient-specific anatomical modeling and printing of such models; these enhancements can even be taken into the OR [11]. This narrative review aims to analyze the current literature on the application of 3D printing technology in both proximal humerus fractures and their sequelae by assessing its use in preoperative planning, intraoperative guidance, and, ultimately, its impact on patient outcomes. Specifically, this study seeks to provide an overview of how 3D technologies are currently integrated into clinical workflows, highlight their role in improving surgical accuracy [12] and decision-making [13], and identify existing limitations and gaps in the evidence base. By examining trends in indications, technical applications [14,15], and reported results, the review also aims to outline future research directions and potential standardization pathways for their use in shoulder trauma and reconstructive surgery.

## 2. Materials and Methods

### 2.1. Data Source and Study Selection

An exhaustive search was conducted across the electronic databases of “Embase”, “Pubmed”, and “Web of Science” for relevant studies employing the following keywords and their combination: “proximal humerus fracture”, “proximal humeral fracture sequelae”, “three-dimensional”, and “3D printing”. The inclusion criteria were limited to English-language studies without specific publication dates. Reference lists of selected articles were searched for any additional articles that were not identified in the database search. Longitudinal studies (retrospective and prospective) that collected data about the use of 3D imaging and printing technology were included. The exclusion criteria included expert opinions, prior systematic reviews, and letters to the editor. Furthermore, we excluded (I) studies that do not address the subject of 3D printing aiding the treatment of fractures and sequelae, like oncologic patients or other kinds of elective surgeries, and (II) studies that included different joints in which shoulder data could not be extrapolated. 

### 2.2. Data Extraction and Standardization

Data extraction included study characteristics (design and the level of evidence), patient demographics (sample size), 3D printing applications, follow-up duration, clinical outcomes, and complications. Data standardization procedures were implemented to address inconsistencies in terminology, including corrections of typographical errors and harmonization of study design classifications, by two authors (M.C. and V.R.), and any disagreement was overseen by a senior author (R.d.G).

### 2.3. Three-Dimensional Printing Application Categories

Studies were classified based on their primary use of 3D printing technology: diagnostic (use for fracture classification and assessment), planning (preoperative surgical planning and templating), intraoperative (surgical guidance and patient-specific instrumentation), and combined (multiple applications within the same study).

### 2.4. Clinical Outcomes, Follow-Up, and Complications

Clinical outcome measures were extracted and standardized using pattern recognition algorithms to identify common assessment tools including ASES, the Constant–Murley score, DASH and QuickDASH, VAS, and range-of-motion measurements. Only studies reporting specific, quantifiable outcome measures were included in the outcome analysis. Follow-up periods were extracted using numerical parsing algorithms and categorized as short-term (<12 months), medium-term (12–24 months), and long-term (≥24 months). Complications were systematically categorized by severity: major (infections, nerve injuries, and implant failures that required revisions), moderate (screw penetration and malposition), minor (pain and stiffness), and none reported. Finally, studies were categorized by sample size to assess statistical power: very small: <10 patients; small: 10–29 patients; medium: 30–99 patients; and large: ≥100 patients.

### 2.5. Statistical Analysis

The SPSS (Statistical Package for the Social Sciences, v20.0, IBM. Armonk, NY, USA) software was used for statistical analysis. Descriptive statistics were calculated for all continuous variables. Categorical data were presented as frequencies and percentages. Cross-tabulation analysis was performed to examine relationships between study types, applications, and outcomes.

## 3. Results

### 3.1. Article Characteristics and Distribution

A total of 63 studies met the inclusion criteria. After further assessment for pertinency to the topic and clarity of outcome data presentation, 19 studies were analyzed, comprising 14 fracture treatment studies (73.7%) and 5 sequela management studies (26.3%). The included studies and main data are summarized in Table 1 and Table 2.

The fracture studies encompassed 636 patients with a mean sample size of 45.4 patients (range: 1–140), while sequelae studies included 28 patients with a mean of 5.6 patients per study (range: 1–20).

### 3.2. Study Design and Evidence Quality

The most prevalent study design in fracture research was case–control retrospective studies (50.0%), followed by case reports (7.1%). Sequela studies were predominantly case reports (60.0%). Level-of-evidence analysis revealed that 78.6% of fracture studies were Level III evidence, while sequela studies showed lower evidence levels with 100% at Levels IV–V.

### 3.3. Three-Dimensional Printing Applications

Preoperative planning represented the most common application of 3D printing technology and was utilized in 57.1% of fracture studies and 80.0% of sequela studies. Diagnostic applications were employed in 35.7% of fracture studies, while intraoperative guidance was more prevalent in sequela management (100% vs. 0% in fractures). Combined applications were observed in 80% of sequela studies but none of the fracture studies.

### 3.4. Clinical Outcome Assessment

Only 42.1% of studies (8/19) reported standardized clinical outcome measures. Range-of-motion assessment was the most frequently used outcome measure (15.8% of studies), followed by the Constant–Murley score (10.5%). The ASES score and DASH questionnaire were utilized in 5.3% and 15.8% of studies, respectively. Fracture studies demonstrated greater diversity in outcome measures compared to sequela studies.

### 3.5. Follow-Up Duration Analysis

Follow-up data was available for 42.1% of studies (8/19). Fracture studies showed significantly longer follow-up periods, with a mean of 18.2 months (range: 12–26.6 months), while sequela studies had a mean follow-up of 18.2 months (range: 2–29 months). Long-term follow-up (≥24 months) was achieved in 33.3% of fracture studies with available data, compared to 60% of sequela studies.

### 3.6. Complication Rates and Safety Profile

Complications were reported or accounted for across all studies. The majority of studies (84.2%) reported no complications. Major complications occurred in 14.3% of fracture studies, including nerve injuries and implant failures, while no major complications were reported in sequela studies. The overall complication rate remained low across both study categories.

### 3.7. Sample Size Distribution and Statistical Power

Sample size analysis revealed significant heterogeneity between study types. Large-scale studies (≥100 patients) comprised 14.3% of fracture studies but were absent in sequela research. Medium-sized studies (30–99 patients) represented 42.9% of fracture studies compared to 0% of sequela studies. Very small studies (<10 patients) constituted 80% of sequela studies versus 21.4% of fracture studies, highlighting the preliminary nature of sequela research in this field.

## 4. Discussion

Across proximal humerus trauma and its sequelae, 3D technologies are transitioning from ad hoc adjuncts to structured workflows that combine segmentation, virtual reduction, physical models, and patient-specific instrumentation (PSI) [32]. Current comparative evidence and umbrella syntheses—albeit heterogeneous—converge on perioperative advantages of 3D-assisted surgery over conventional planning [33], including a shorter operative time, less blood loss, and in some series fewer complications; within the subset of proximal humerus fractures, meta-analytic data also indicate higher early functional scores. Hu et al. have shown that 3D-printed custom implants can provide an effective solution for complex reconstructive scenarios, such as locked posterior shoulder dislocations with reverse Hill–Sachs lesions, enabling precise anatomical restoration when conventional techniques are insufficient [29]. These gains likely reflect improved preoperative understanding of fragment morphology and implant trajectories, rehearsal of reduction/osteosynthesis on life-size replicas, and translation of plans via PSI, as mentioned by Fidanza et al. [34]. In fracture sequelae, where deformity is multiplanar and the bone stock is altered, early prospective and cohort experiences suggest that 3D planning with PSI can standardize corrective osteotomies [35]—often preceding or combined with reverse shoulder arthroplasty—achieving clinically meaningful short- to mid-term improvements with acceptable complication profiles [10]. Delbrück et al. investigated corrective osteotomies of complex upper-extremity deformities using patient-specific instruments and showed good mid-term clinical outcomes with high plan-to-postoperative 3D concordance, highlighting that analyzing sources of error across the workflow (from CT planning to execution) is essential to optimize accuracy and identify correlation with clinical results [36]. From a diagnostic and decision-support standpoint, randomized and observational studies indicate that handling 3D-printed models can increase inter-rater agreement for AO/OTA and Neer classifications and improve concordance on treatment indication compared with CT alone—particularly among shoulder surgeons—thereby supporting the use of tangible models in education, MDT discussions, and informed consent, as studied by Bougher et al. [2]. Notably, other well-designed investigations found no improvement in recognizing several granular fracture features when 3D models were added to standard imaging, underscoring that diagnostic benefits are not universal and may depend on user expertise and the task. In detail, Berhouet et al. demonstrated that in reverse shoulder arthroplasty, the actual postoperative range of motion often deviates significantly from the preoperative virtual plan, particularly for elevation and rotation movements. This discrepancy highlights the current limitations of static simulation models in accurately replicating real postoperative function, which may be influenced by multiple intraoperative factors such as soft-tissue tension, implant positioning tolerances, and patient-specific anatomy. Their findings underscore the need to refine and validate preoperative 3D planning workflows to ensure more reliable prediction of functional outcomes in shoulder reconstruction [37]. Similarly, Luxenhofer et al. investigated the diagnostic accuracy of intraoperative 3D imaging in complex articular fractures, demonstrating that the use of high-resolution intraoperative CT can significantly improve the detection of residual incongruities compared with conventional fluoroscopy. This enhanced visualization allows for immediate intraoperative corrections, effectively narrowing the gap between virtual preoperative planning and real-time surgical execution. Their results reinforce the role of advanced 3D imaging as a critical tool to ensure that the planned reconstruction is achieved with higher precision and reproducibility in complex fracture management [38]. Despite these promising signals, important limitations persist. Most clinical studies remain single-center, small, and observational (Levels III–V), with incomplete standardization of functional outcomes, radiation metrics, and cost reporting [17,39]. Technical cautions also emerge: planned versus implanted hardware can diverge at surgery, and upper-limb PSI workflows report pitfalls such as guide mal-seating [16], error propagation from pre-drilled holes [35], translation across oblique osteotomy planes, and depth estimation challenges near cartilage—issues directly relevant to the proximal humerus [40]. For instance, Chen et al. highlighted several practical limitations of patient-specific instrumentation, noting that even with careful planning, guide placement can be challenging, sometimes leading to malpositioning or a poor fit on the bone surface. They also observed that maintaining reduction during fixation can be difficult and that the accuracy of PSI ultimately depends as much on intraoperative handling as on the quality of the preoperative plan [16]. These data argue for robust intraoperative verification and thoughtful indication rather than indiscriminate adoption [41]. Future directions should include multi-center RCTs comparing 3D-assisted fixation and reconstruction against purely virtual planning and other digital navigational approaches (e.g., augmented reality/virtual reality), with harmonized reporting of ASES/CMS/DASH, complications, reoperation, radiation, and time/cost endpoints [8]. Economic analyses—already suggesting shorter procedures and procedure-level savings in PHF—require replication at scale with payer perspectives. Finally, as segmentation and templating mature, automation and AI-assisted simulations could deliver reproducible, generalizable protocols for PHF and sequelae, moving 3D planning from craft to standard of care where it demonstrably improves outcomes. Italia et al. explored the use of advanced 3D planning and mixed reality guidance in single-stage revision reverse shoulder arthroplasty, showing how extended visualization can support more accurate implant positioning and help navigate complex revision cases, and their findings suggest that combining virtual planning with intraoperative immersive tools can make surgical execution more consistent, though its real impact on outcomes still needs to be better defined [42].

Our study has its limitations. Specifically, the retrospective nature and reliance on the existing literature introduces inherent biases, and the variability in reporting standards across included studies complicates the direct comparison and synthesis of findings. Moreover, the exclusion of unpublished data or studies not available in English might have introduced publication and language biases, potentially skewing the overall landscape of evidence [43].

## 5. Conclusions

The evidence emerging from this review highlights the evolving role of three-dimensional technologies in the management of proximal humerus fractures and their sequelae. In the setting of acute trauma, 3D printing and virtual planning have reached a stage of clinical maturity, with consistent evidence supporting their utility in improving fracture understanding, surgical planning, and classification accuracy. These technologies facilitate more precise visualization of fracture morphology, allowing for tailored surgical strategies and potentially contributing to more reproducible intraoperative decision-making. By contrast, their use in the management of post-traumatic deformities remains at an earlier stage of development, reflecting the greater complexity of these cases. Sequelae often involve multiplanar deformities, distorted anatomy, and poor bone stock, which pose unique technical challenges for corrective osteotomy and prosthetic reconstruction. Current evidence in this field is largely based on small case series and reports, limiting the generalizability of findings. However, early results suggest that 3D planning and patient-specific instrumentation can offer meaningful advantages in terms of accuracy and individualized treatment. Across both settings, a consistent gap remains between preoperative virtual planning and intraoperative execution, underscoring the need for workflow refinement and integration with real-time imaging or mixed reality technologies. Future research should prioritize larger, multicenter prospective studies with standardized outcome measures, cost-effectiveness analyses, and robust evaluation of clinical endpoints. Establishing shared protocols could help translate these promising technologies from specialized centers into broader clinical practice, ultimately improving the quality and precision of shoulder trauma and reconstructive surgery [44].

## Figures and Tables

**Table 1:** List and characteristics of studies on the use of 3D technology in proximal humerus fractures. (AO = Arbeitsgemeinschaft für Osteosynthesefragen—classification for humeral fractures; CMS = Constant–Murley score; DASH = Disability of the Arm, Shoulder, and Hand score; ASES = American Shoulder and Elbow Surgeons score).

Authors	Study Design	Patients (n)	Follow-Up (Months)	Classification Used	3D Application	Clinical Outcome	Complications
Bougher et al. [2]	Case–Control Study/Science-Based Study	30	73 (49–96)	NEER	DIAGNOSTIC	-	-
Chen et al. [16]	Case–Control Study Therapeutic	32	69.5 (49–86)	NEER	PLANNING	ASES, CMS, and SF-36	1 Screw Penetration, 1 Implant Failure, and 2 Infections
Cocco et al. [17]	Observational Cross-Sectional Study	75	-	AO; NEER	DIAGNOSTIC AND TREATMENT	-	-
Cocco et al. [18]	Observational Cross-Sectional Study	9	-	AO; NEER	DIAGNOSTIC	-	-
Hu et al. [19]	Case–Control Retrospective	21	50.05 ± 12.75	AO	PLANNING	qDash and CMS	2 Radial Nerve Palsies
Khanna et al. [20]	Case–Control Retrospective	28	-	AO	PLANNING	-	-
Kim et al. [21]	Science-Based Study/Case Series	30	46.3 ± 16.4 Years (20–74)	-	PLANNING	-	-
Poltaretskyi et al. [22]	Case Series	57	66 Years (23–87)	-	DIAGNOSTIC AND TREATMENT	-	-
Puglisi et al. [23]	Case–Control Retrospective	9	68 Years (54–74)	AO; NEER; HERTEL	DIAGNOSTIC AND TREATMENT	-	-
Qiang et al. [24]	Case–Control Retrospective	134	49.5 ± 9.8 Years (22–77 Years)	-	PLANNING	-	-
Russo et al. [25]	Basic Science Study/Case–Control Retrospective	50	62 (18–90)	-	CLASSIFICATION	-	-
Spek et al. [26]	Case–Control Retrospective Diagnostic	20	-	HERTEL; NEER	PLANNING	-	-
Thati et al. [27]	Case Report	1	58	-	PLANNING	Range of Motion	1 Screw Penetration
Vlachopoulos et al. [28]	Basic Science Study Case–Control Retrospective	140	CADAVERS	-	PLANNING	-	-

**Table 2:** List and characteristics of studies on the use of 3D technology in proximal humerus fracture sequelae. (RSA = Reverse Shoulder Arthroplasty; TSA = Total Shoulder Arthroplasty; CMS = Constant–Murley score; DASH = Disability of the Arm, Shoulder, and Hand score).

Authors	Type of Study	Patients (n)	Follow-Up (Months)	Follow-Up (Months)	Classification Used	3D Application	Prosthesis Type (n)	Clinical scores	Valore (Range)	Complications
Hu et al. [29]	Case Report	1	60	2	-	IntraOp	Hemi	VAS	2	-
Cozzolino et al. [30]	Prospective Cohort Study	20	69.7	24	Boileau	Planning; IntraOp	RSA, TSA	CMS, VAS, DASH	67.7; 1.6; 24.1	1 infection
Russo et al. [10]	Surg. Tech. and Case Report	1	70	29	-	Planning; IntraOp	RSA	CMS; DASH	46; 59.2	-
Thati et al. [27]	Case Report	1	58	12	-	Planning; IntraOp	RSA	-	-	-
Mothes et al. [31]	Case Series	5	42.7	24	-	Planning; IntraOp	RSA	-	-	-

## References

[1] Jabran A., Peach C., Ren L. (2018). Biomechanical Analysis of Plate Systems for Proximal Humerus Fractures: A Systematic Literature Review. Biomed. Eng. Online.

[2] Bougher H., Büttner P., Smith J.F.D., Banks J., Na H.S., Forrestal D.P., Heal C. (2020). Interobserver and Intraobserver Agreement of Three-Dimensionally Printed Models for the Classification of Proximal Humeral Fractures. JSES Int..

[3] Malfi P., de Giovanni R., Bernasconi A., Rossi V., Grasso R., Cozzolino A. (2024). Reverse Shoulder Arthroplasty for Two-Parts Proximal Humerus Fractures with “Shish-Kebab” Technique. JSES Rev. Rep. Tech..

[4] Li K., Liu Z., Li X., Wang J. (2022). 3D Printing-Assisted Surgery for Proximal Humerus Fractures: A Systematic Review and Meta-Analysis. Eur. J. Trauma Emerg. Surg..

[5] Cozzolino A., Malfi P., de Giovanni R., Fedele A., Rusconi G., Guarino A., Pietto F.D., Russo R. (2023). Computed Tomography Improves the Diagnostic Accuracy but Not the Interobserver Reliability of the Boileau Classification of Proximal Humerus Fracture Sequelae. Shoulder Elb..

[6] de Giovanni R., Malfi P., Mottola L., Giordano E., Hasler J., Zumstein M.A., Cozzolino A. (2025). Which Is the Optimal Treatment and the Most Significant Prognostic Factor for Reverse Shoulder Arthroplasty in Case of PHAROS 2 Humeral Bone Loss? A Proportional Meta-Analysis of Non-Oncologic Patients. JSES Int..

[7] Wang J., Cai L., Xie L., Chen H., Guo X., Yu K. (2019). 3D Printing-Based Ganz Approach for Treatment of Femoral Head Fractures: A Prospective Analysis. J. Orthop. Surg. Res..

[8] Tueni N., Amirouche F. (2025). Branding a New Technological Outlook for Future Orthopaedics. Bioengineering.

[9] Moldovan F., Gligor A., Bățagă T. (2021). Structured Integration and Alignment Algorithm: A Tool for Personalized Surgical Treatment of Tibial Plateau Fractures. J. Pers. Med..

[10] Russo R., Cozzolino A., Guastafierro A., Rotonda G.D., Viglione S., Ciccarelli M., Mortellaro M., Minopoli P., Fiorentino F., Pietroluongo L.R. (2021). Use of 3D Planning and Patient-Specific Guides for Proximal Humerus Corrective Osteotomy Associated With Shoulder Prosthesis Implantation in Proximal Humeral Varus Malunion. Tech. Hand Up Extrem. Surg..

[11] Baburaj V., Patel S., Kumar V., Dhillon M.S. (2022). Utility of 3D Printing in the Surgical Management of Intra-Articular Distal Humerus Fractures: A Protocol for Systematic Review and Meta-Analysis. MedRxiv.

[12] Beliën H., Biesmans H., Steenwerckx A., Bijnens E., Dierickx C. (2017). Prebending of Osteosynthesis Plate Using 3D Printed Models to Treat Symptomatic Os Acromiale and Acromial Fracture. J. Exp. Orthop..

[13] Wu R., Zhang W., Lin Y.-Z., Fang Z.-L., Wang K., Wang C., Yu D. (2023). Influence of Preoperative Simulation on the Reduction Quality and Clinical Outcomes of Open Reduction and Internal Fixation for Complex Proximal Humerus Fractures. BMC Musculoskelet. Disord..

[14] Brouwer K.M., Lindenhovius A., Dyer G.S.M., Zurakowski D., Mudgal C.S., Ring D. (2012). Diagnostic Accuracy of 2- and 3-Dimensional Imaging and Modeling of Distal Humerus Fractures. J. Shoulder Elb. Surg..

[15] You W., Liu L.J., Chen H.X., Xiong J., Wang D.M., Huang J., Ding J., Wang D.P. (2016). Application of 3D Printing Technology on the Treatment of Complex Proximal Humeral Fractures (Neer3-Part and 4-Part) in Old People. Orthop. Traumatol. Surg. Res..

[16] Chen Y., Jia X., Qiang M., Zhang K., Chen S. (2018). Computer-Assisted Virtual Surgical Technology Versus Three-Dimensional Printing Technology in Preoperative Planning for Displaced Three and Four-Part Fractures of the Proximal End of the Humerus. J. Bone Jt. Surg..

[17] Cocco L.F., Aihara A.Y., Franciozi C.E.d.S., dos Reis F.B., Luzo M.V.M. (2020). Three-Dimensional Models Increase the Interobserver Agreement for the Treatment of Proximal Humerus Fractures. Patient Saf. Surg..

[18] Cocco L.F., Yazzigi J.A., Kawakami E.F.K.I., Alvachian H.J.F., dos Reis F.B., Luzo M.V.M. (2019). Inter-Observer Reliability of Alternative Diagnostic Methods for Proximal Humerus Fractures: A Comparison between Attending Surgeons and Orthopedic Residents in Training. Patient Saf. Surg..

[19] Hu C., Qiu B., Cen C., Luo Q., Cao Y. (2024). 3D Printing Assisted MIPO for Treatment of Complex Middle-Proximal Humeral Shaft Fractures. BMC Musculoskelet. Disord..

[20] Khanna K., Brabston E.W., Qayyum U., Gardner T.R., Levine W.N., Jobin C.M., Ahmad C.S. (2018). Proximal Humerus Fracture 3-D Modeling. Am. J. Orthop..

[21] Kim H., Chung Y., Jang J.S., Kim Y., Park S.B., Song H.S. (2020). Why Locking Plates for the Proximal Humerus Do Not Fit Well. Arch. Orthop. Trauma Surg..

[22] Poltaretskyi S., Chaoui J., Mayya M., Hamitouche C., Bercik M., Boileau P., Walch G. (2017). Prediction of the Pre-Morbid 3D Anatomy of the Proximal Humerus Based on Statistical Shape Modelling. Bone Jt. J..

[23] Puglisi G., Montemagno M., Denaro R., Condorelli G., Caruso V., Vescio A., Testa G., Pavone V. (2022). 3D-Printed Models versus CT Scan and X-Rays Imaging in the Diagnostic Evaluation of Proximal Humerus Fractures: A Triple-Blind Interobserver Reliability Comparison Study. Adv. Orthop..

[24] Qiang M., Jia X., Chen Y., Zhang K., Li H., Chen J., Zhang Y. (2018). Assessment of Screw Length of Proximal Humerus Internal Locking System (PHILOS) Plate for Proximal Humeral Fractures Using Three-Dimensional Computed Tomography Scan. Med. Sci. Monit..

[25] Russo R., Guastafierro A., Rotonda G.D., Viglione S., Ciccarelli M., Mortellaro M., Minopoli P., Fiorentino F., Pietroluongo L.R. (2020). A New Classification of Impacted Proximal Humerus Fractures Based on the Morpho-Volumetric Evaluation of Humeral Head Bone Loss with a 3D Model. J. Shoulder Elb. Surg..

[26] Spek R.W.A., Schoolmeesters B., Oosterhoff J.H.F., Doornberg J.N., van den Bekerom M.P.J., Jaarsma R.L., Eygendaal D., IJpma F.F.A. (2021). 3D-Printed Handheld Models Do Not Improve Recognition of Specific Characteristics and Patterns of Three-Part and Four-Part Proximal Humerus Fractures. Clin. Orthop. Relat. Res..

[27] Thati B., Bodanki C., Badam V.K., Reddy M.V., Reddy A.V.G. (2020). Custom 3D Printed Jigs in Salvage Reverse Shoulder Arthroplasty for Failed Four-Part Proximal Humerus Fracture Fixation: A Case Report. PubMed.

[28] Vlachopoulos L., Dünner C., Gass T., Graf M.J., Göksel O., Gerber C., Székely G., Fürnstahl P. (2015). Computer Algorithms for Three-Dimensional Measurement of Humeral Anatomy: Analysis of 140 Paired Humeri. J. Shoulder Elb. Surg..

[29] Hu Y., Yang K., Liu F., Wang L., Wang S., Zhang X., Qü B., Yang H. (2023). 3D-Printed Custom Implant for the Management of “Locked” Posterior Dislocation of the Shoulder Joint with Reverse Hill-Sachs Lesion: A Case Report. Front. Bioeng. Biotechnol..

[30] Cozzolino A., Guastafierro A., Bernasconi A., Della Rotonda G., Malfi P., Fedele A., Mortellaro M., Minopoli P., Renata Pietroluongo L., Russo R. (2023). Proximal Humerus Fracture Sequelae: Are Corrective Osteotomies Still a Taboo? The Role of Three-Dimensional Preoperative Planning and Patient-Specific Surgical Guides for Proximal Humerus Corrective Osteotomy in Combinazion with Reverse Shoulder Arthroplasty. JSES Int..

[31] Mothes F.C., Britto A.G., Matsumoto F., Tonding M., Ruaro R. (2018). Application of Three-Dimensional Prototyping in Planning the Treatment of Proximal Humerus Bone Deformities. Rev. Bras. Ortop. (Engl. Ed.).

[32] Yasen Z., Robinson A., Woffenden H. (2024). Advanced Preoperative Planning Techniques in the Management of Complex Proximal Humerus Fractures. Cureus.

[33] Wang Q., Hu J., Guan J., Chen Y., Wang L. (2018). Proximal Third Humeral Shaft Fractures Fixed with Long Helical PHILOS Plates in Elderly Patients: Benefit of Pre-Contouring Plates on a 3D-Printed Model—A Retrospective Study. J. Orthop. Surg. Res..

[34] Fidanza A., Caggiari G., Petrillo F.D., Fiori E., Momoli A., Logroscino G. (2024). Three-Dimensional Printed Models Can Reduce Costs and Surgical Time for Complex Proximal Humeral Fractures: Preoperative Planning, Patient Satisfaction, and Improved Resident Skills. J. Orthop. Traumatol..

[35] Lin X., Tang P., Yang S., Su J., Ma W., Tan H., Zhu Y., Xiao W., Wen T., Li Y. (2025). Comparing the Efficacy of 3D-Printing-Assisted Surgery with Traditional Surgical Treatment of Fracture: An Umbrella Review. J. Orthop. Traumatol..

[36] Delbrück H., Weber C.D., Eschweiler J., Hildebrand F. (2022). 3D Accuracy and Clinical Outcomes of Corrective Osteotomies with Patient-Specific Instruments in Complex Upper Extremity Deformities: An Approach for Investigation and Correlation. Eur. J. Med. Res..

[37] Berhouet J., Samargandi R., Favard L., Turbillon C., Jacquot A., Gauci M.-O. (2023). The Real Post-Operative Range of Motion Differs from the Virtual Pre-Operative Planned Range of Motion in Reverse Shoulder Arthroplasty. J. Pers. Med..

[38] Luxenhofer M., Beisemann N., Schnetzke M., Vetter S.Y., Grützner P.A., Franke J., Keil H. (2020). Diagnostic Accuracy of Intraoperative CT-Imaging in Complex Articular Fractures—A Cadaveric Study. Sci. Rep..

[39] Via G.G., Brueggeman D.A., Lyons J.G., Ely I.C., Froehle A.W., Krishnamurthy A. (2022). Funding Has No Effect on Clinical Outcomes of Total Joint Arthroplasty Emerging Technologies: A Systematic Review of Bibliometrics and Conflicts of Interest. Arthroplasty.

[40] Campana V., Cardona V., Vismara V., Monteleone A.S., Piazza P., Messinese P., Mocini F., Sircana G., Maccauro G., Saccomanno M.F. (2020). 3D Printing in Shoulder Surgery. Orthop. Rev..

[41] Liang H., Guo W., Yang Y., Li D., Yang R., Tang X., Yan T. (2022). Efficacy and Safety of a 3D-Printed Arthrodesis Prosthesis for Reconstruction after Resection of the Proximal Humerus: Preliminary Outcomes with a Minimum 2-Year Follow-Up. BMC Musculoskelet. Disord..

[42] Italia K., Launay M., Gilliland L., Nielsen J., Pareyón R., Hollman F., Salhi A., Maharaj J., Jomaa M., Cutbush K. (2022). Single-Stage Revision Reverse Shoulder Arthroplasty: Preoperative Planning, Surgical Technique, and Mixed Reality Execution. J. Clin. Med..

[43] Wah J.N.K. (2025). The Rise of Robotics and AI-Assisted Surgery in Modern Healthcare. J. Robot. Surg..

[44] Moolenaar J.Z., Tümer N., Checa S. (2022). Computer-Assisted Preoperative Planning of Bone Fracture Fixation Surgery: A State-of-the-Art Review. Front. Bioeng. Biotechnol..

